# Management and outcome of pediatric metastatic Wilms’ tumor at the National Cancer Institute, Egypt

**DOI:** 10.1186/s43046-020-00031-7

**Published:** 2020-04-15

**Authors:** Moatasem Elayadi, Sarah Magdy, Ehab Khalil, Wael Zekri

**Affiliations:** 1 Department of Pediatric Oncology, National Cancer Institute, Cairo University, El-khalig Square, Kasr El-Aini St., Cairo, 11796 Egypt; 2 Department of Radiation Oncology, National Cancer Institute, Cairo University, Cairo, Egypt

**Keywords:** Wilms’ tumor, Nephroblastoma, Metastatic, Stage IV, Pediatric

## Abstract

**Background:**

Wilms’ tumor (WT) is the most common renal malignant tumor of childhood. Metastatic WT has a worse prognosis than localized disease. This study aims to assess the clinical outcome and different prognostic factors that influence treatment outcome of pediatric metastatic WT cases treated at National Cancer Institute (NCI), Egypt, between January 2008 and December 2015. Medical records were retrospectively reviewed for clinical, radiological and histopathological data, treatment received, and survival outcome.

**Results:**

In the specified study period, 24/103 (23.3%) patients with WT were metastatic at presentation. The mean age was 5.25 ± 2.87 years (range 2.0–12.7). Abdominal swelling/mass was the commonest presentation (70.8%). Only 3 patients (12.5%) had combined lung and liver metastases while 21 patients (87.5%) had pulmonary-only metastases. All patients had favorable histology tumors with no anaplasia. Nine patients (37.5%) underwent upfront nephrectomy. Majority of patients (91.7%) had local stage III disease. Surgical complications were reported in 4 patients; 3 of them had up-front nephrectomy. Only 7/21 patients achieved rapid complete response of pulmonary nodules after 6 weeks of chemotherapy (CTH), and they had a better survival outcome. Patients were followed up till December 2017. Thirteen patients (54.1%) experienced events during the study period including 5 relapses, 6 cases with disease progression, and 2 patients died out of sepsis. The 3-year event-free and overall survival rates were 48.2% and 54.2%, respectively.

**Conclusion:**

Neo-adjuvant CTH followed by delayed nephrectomy seems more suitable approach in our institute. Pulmonary response to neo-adjuvant CTH appears to be a strong predictor for outcome.

## Background

Nephroblastoma or Wilms’ tumor (WT) is the most common renal malignant tumor of childhood. Approximately 12% of WT are metastatic at presentation, with 80% having pulmonary metastases [1]. The primary distant site for WT metastases is the lungs, while hepatic metastases are much less common [2]. Stage IV disease has the worst outcome between the favorable histology WT and severe short and long-term morbidity due to the more intensive chemotherapy used and also due to the irradiation to both the metastatic sites and the tumor bed [3]. Response of pulmonary nodules to neo-adjuvant chemotherapy (CTH) is an important factor to predict patients’ outcome [1]. The purpose of this study is to assess the clinical outcome of WT patients with metastases at initial presentation and to study the different factors that may influence their prognosis.

## Methods

This is a retrospective study, including all newly diagnosed pediatric patients with metastatic unilateral WT from 1^st^ January, 2008 to 31^st^ December, 2015. Records of all patients were reviewed for details of demographic data, initial investigations; CT scans and histopathology, revision of staging, treatment received, and response to treatment.

All patients had CT chest and abdomen with contrast as initial work-up for detection of pulmonary or hepatic metastases. Assessment of treatment response was done after 6 weeks of neo-adjuvant chemotherapy and was discussed through multidisciplinary team (MDT) meetings. All CT films were reviewed by experts in the radio-diagnosis department indicating the metastatic nature of the lung or hepatic lesions. The pathology assessment and determination of favorable vs unfavorable histology followed the guidelines of COG protocol [4, 5].

**Fig. 1:**
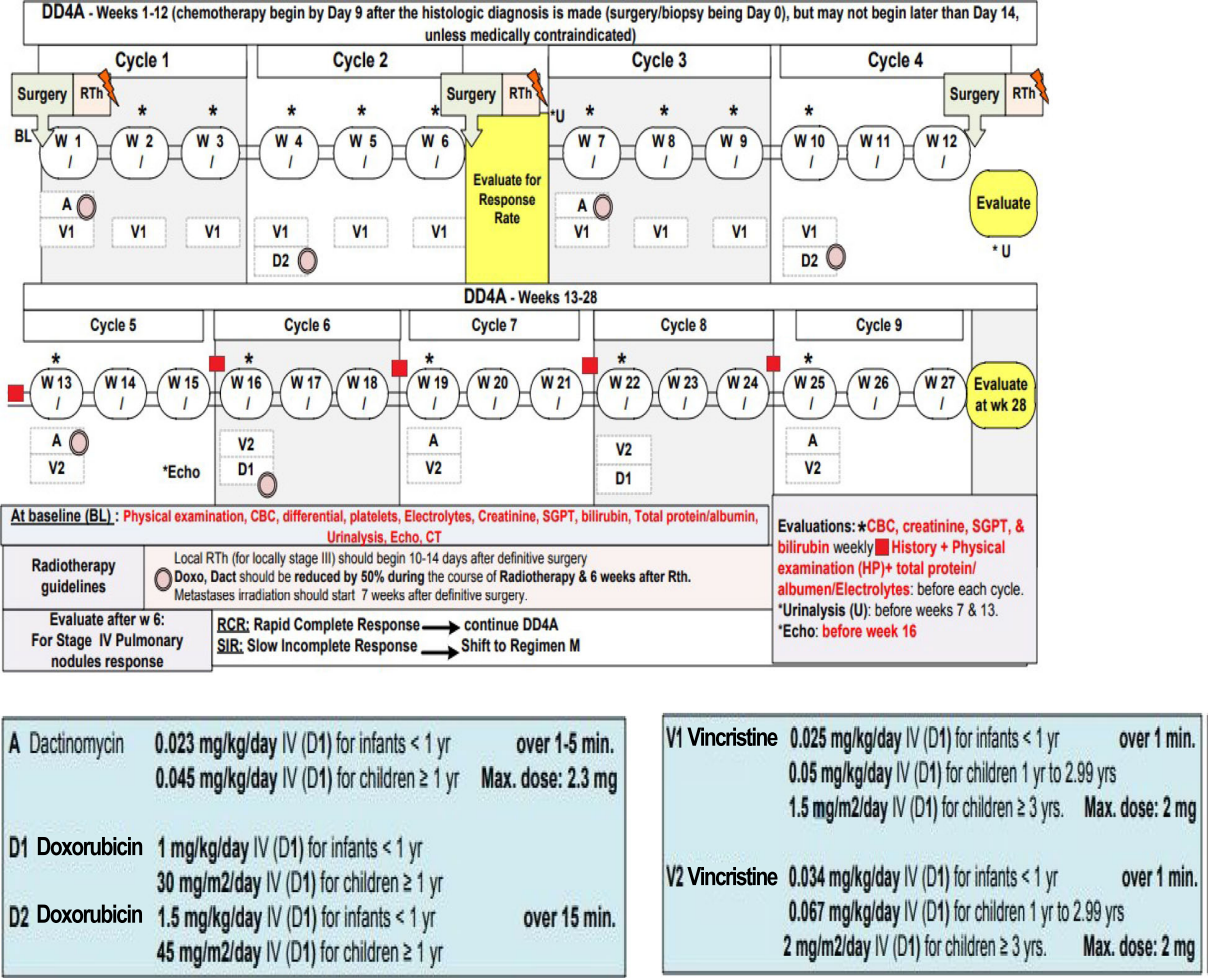
Regimen DD-4A

**Fig. 2:**
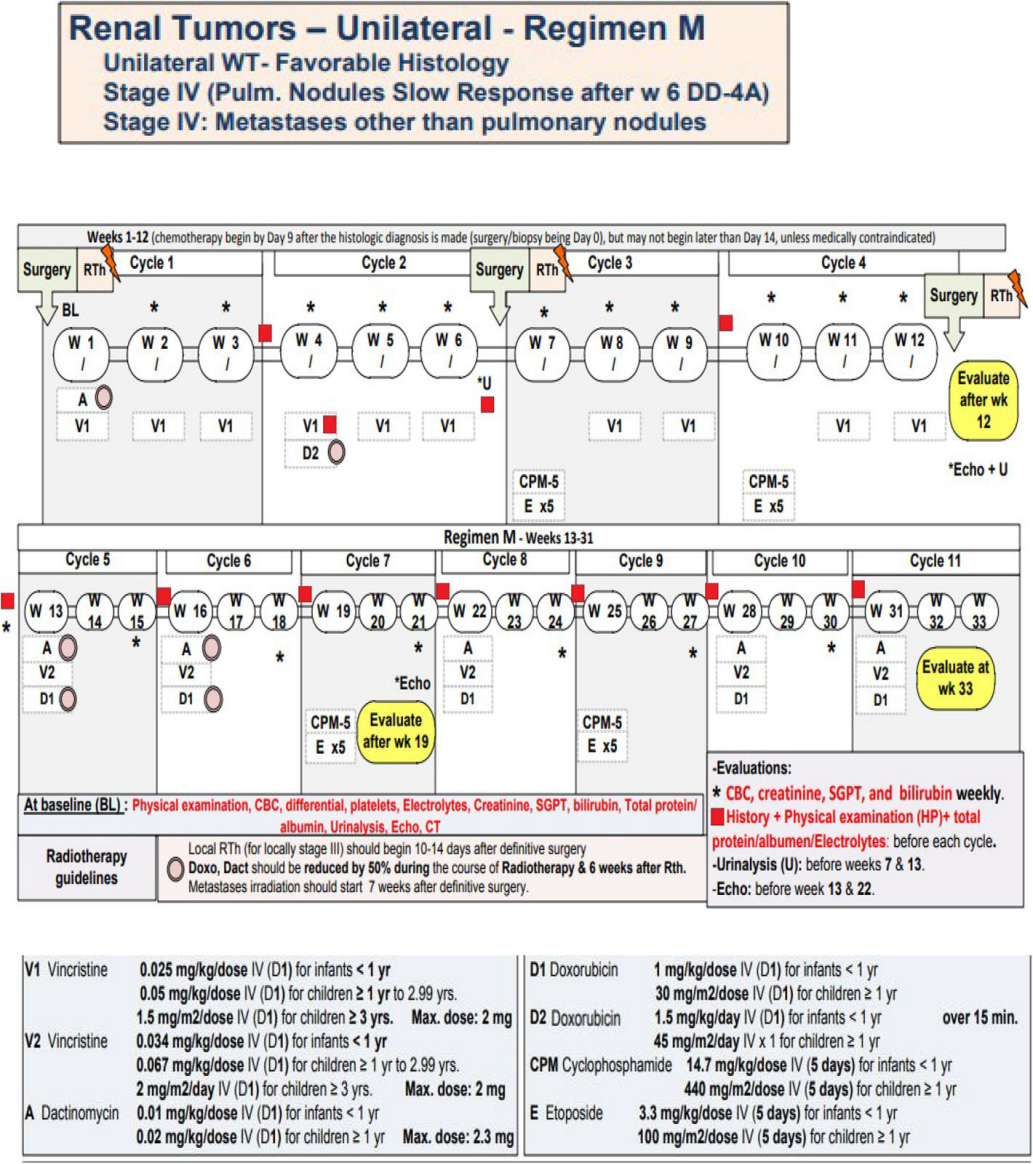
Regimen M

Patients were treated according to modified COG protocol “AREN0533” (Figs. 1 and 2) where they received 3-drug regimen (regimen DD-4A) or 5-drug regimen (regimen M). Regimen DD-4A consists of vincristine, doxorubicin, and dactinomycin, while regimen M consists of the same 3 drugs as DD4A regimen plus cyclophosphamide and etoposide (for doses, refer to Figs. 1 and 2). Unlike the original “AREN0533” protocol, loss of heterozygosity (LOH) status for 1p and 16q chromosomes was not included in risk stratification for enrolled patients.

Patients were followed up till 31^st^ December 2017. Different prognostic factors were analyzed and correlated with outcome, including the site of metastasis (pulmonary, extra pulmonary, or both), initial management (CTH or upfront nephrectomy), local stage, histology, timing of radiotherapy (RT), and pulmonary response to CTH in terms of rapid complete response (RCR) or slow incomplete response (SIR).

Overall survival (OS) was defined as the time between date of diagnosis and date of death, from any cause, or the date of last visit. Event-free survival (EFS) was defined as the time from date of diagnosis to date of progression, relapse, death, or last follow-up, whichever occurred first. Statistical analysis was done using IBM© SPSS© Statistics version 22 (IBM© Corp., Armonk, NY, USA). Numerical data were expressed as mean and standard deviation or median and range as appropriate. Qualitative data were expressed as frequency and percentage. Survival analysis was done using Kaplan-Meier method, and comparison between two survival curves was done using log-rank test. All tests were two-tailed. A *p* value < 0.05 was considered significant.

## Results

### Patients’ characteristics

During the study period, 24 out of 103 patients with WT (23.3%) were metastatic at initial presentation to NCI. The mean age at diagnosis was 5.25 ± 2.87 years (range 2.0 to 12.7 years). Females constituted 70.8% (17/24), with a male to female ratio of 1:2.4. The primary site of disease was the left kidney in 16/24 patients (66.7%). Abdominal swelling/mass was the commonest presentation (17 patients, 70.8%) (Table 1). No congenital anomalies/syndromes were detected in the study patients. All patients were diagnosed to have pulmonary nodules by CT chest, and only 3 patients (12.5%) had combined multiple lung and liver metastases at initial presentation.

**Table 1:** Demographic data and clinical characteristics of the study group (*n* = 24)

Age at diagnosis (years)	
Mean ± SD	5.25 ± 2.87
(Range)	(2.0–12.7)
Gender	
Male	7 (29.2%)
Female	17 (70.8%)
Laterality of tumor	
Right	8 (33.3%)
Left	16 (66.7%)
IVC thrombus	
Yes	3 (12.5%)
No	21 (87.5%)
Presenting symptoms	
Abdominal swelling	17 (70.8%)
Hematuria	5 (20.8%)
Abdominal pain	3 (12.5%)
Asymptomatic	1 (4.2%)
Site of metastases	
Lung	21 (87.5%)
Lung and liver	3 (12.5%)

### Initial therapy

**Fig. 3:**
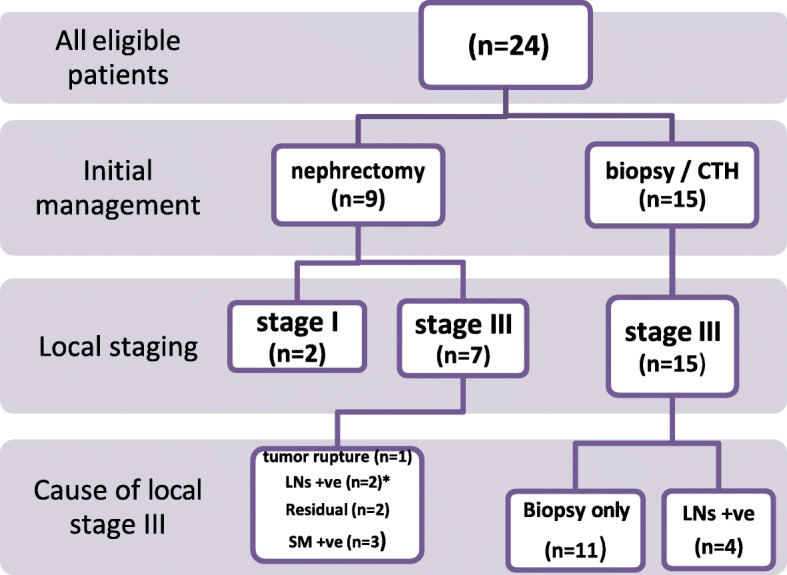
Initial management and local staging of study patients. CTH, chemotherapy; SM, surgical margin; LNs, lymph nodes. *: including the patient with tumor rupture

All study patients initially received 3-drug regimen (DD-4A) except for one patient with both lung and liver metastases who received 5-drug regimen (regimen M). All CT abdomen were reviewed initially by our surgeons to take the decision either to go for surgery or to start neo-adjuvant chemotherapy. Up-front nephrectomy was done for 9 patients (37.5%), and 7/9 patients (77.7%) had stage III local disease because of presence of post-operative residual (*n* = 2, 28%), surgical margin positive for infiltration (*n* = 3, 42%), LN involvement (*n* = 2, 28%), and intra-operative tumor rupture (*n* = 1, 14%) (Table 2 and Fig. 3). All remaining patients (*n* = 15, 62.5%) started neo-adjuvant CTH and showed regressive course of their tumors in CT evaluation post week 6 except for 1 patient whose CT showed a stationary course of the tumor and was shifted to 2^nd^ line CTH.

**Table 2:** Surgical and pathological outcome of patients with up-front nephrectomy (*n* = 9)

Patient no.	Surgical complications	Post-op residual	Pathology		Local stage	Local residual @ W (6)
			SM	LNs		
1	+ve	Not assessed	+ve	−ve	3	−ve
2	−ve	Not assessed	+ve	Not assessed	3	+ve
3	−ve	Not assessed	+ve	Not assessed	3	+ve
4	−ve	+ve	−ve	−ve	3	−ve
5	−ve	+ve	−ve	Not assessed	3	−ve
6	−ve	−ve	−ve	−ve	1	−ve
7	+ve	−ve	−ve	+ve	3	−ve
8	+ve	−ve	−ve	+ve	3	−ve
9	−ve	−ve	−ve	−ve	1	−ve

Twenty-one patients had metastasis in their lungs only, and they were assessed for pulmonary response at week 6. Seven patients (33.3%) achieved RCR, showing complete resolution of pulmonary nodules by chemotherapy only. There was no surgical intervention for the residual lung nodules after 6 weeks of neo-adjuvant CTH; this is because chest operations are difficult to do together with so much delay of recovery and the possibility of some complications; meanwhile, the revision of the CT chests with our radio-diagnosis departments and whether these residual lesions were at the same sites of the original lesions was the main method of diagnosing rapid vs delayed pulmonary responses.

Surgical complications occurred in 4 patients; 3 patients (33.0%) had up-front nephrectomy, and one patient (6.0%) underwent delayed nephrectomy after neo-adjuvant CTH. These complications were intra-operative tumor rupture (*n* = 1), pulmonary embolism (*n* = 1), and intestinal obstruction (*n* = 2).

All nephrectomy specimens revealed favorable histology with no evidence of anaplasia. Pathology of delayed nephrectomy of 2 patients revealed blastemal predominance. All cases had negative surgical and vascular margins except for 3 patients from the up-front nephrectomy group who had their surgical margins positive for infiltration with tumor cells. None of the 3 patients had early post-operative imaging; however, 2 of them had a gross residual disease in CT evaluation at week 6 (Table 2).

### Adjuvant therapy

#### Radiotherapy

Local RT was given to 21/24 patients (87.5%). Three patients were deferred from irradiation; one patient had local stage I disease after up-front nephrectomy, and 2 patients had progressive disease before local control. Flank RT was implemented to 16/21 patients, and all of them had local stage III. Five patients were treated by whole abdominal irradiation (WAI).

Seven patients achieved pulmonary RCR post neo-adjuvant CTH, and 5/7 patients (71.4%) were spared lung irradiation. All patients with pulmonary SIR (*n* = 14) were treated by lung RT except for 3 patients; one with residual tiny nodule (5 mm in diameter) and 2 patients developed local progression of their tumors while on treatment. Patients with both lung and liver metastases (*n* = 3) were treated by RT to the lung and liver. For all patients treated with RT, median time between surgery and start of RT was 19 days (range 4–208 days). Only 7/21 patients (33.3%) had their RT within 14 days from date of surgery.

#### Chemotherapy

Patients with pulmonary RCR (*n* = 7) continued the same 3-drug regimen (DD-4A). Fourteen patients had pulmonary SIR; 10/14 patients were changed to 5-drug regimen, while 4 patients continued the same 3-drug regimen. Three patients had both lung and liver metastases at presentation; one patient was treated by 5-drug regimen, second patient started on 3-drug regimen then was changed to 5-drug regimen after delayed nephrectomy post week 6 while the third patient was treated by 3-drug regimen all through.

### Events and survival rates

**Fig. 4:**
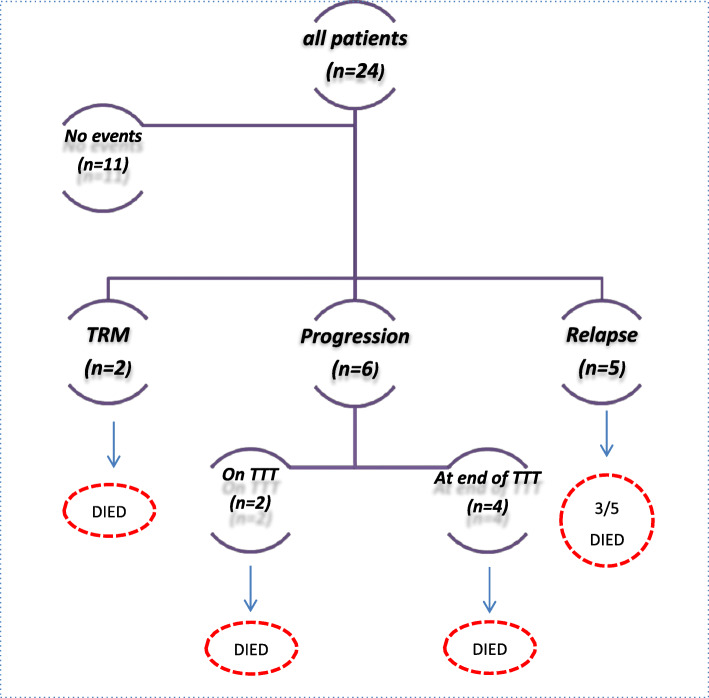
Events among study cohort. TRM, treatment related mortality; TTT, treatment

Thirteen patients (54.1%) experienced events during the study period; 2 patients (15.4%) had complicated episodes of febrile neutropenia and died out of sepsis while on treatment; 5 patients (38.5%) had disease relapse, and 6 patients (46.1%) had disease progression. Relapse sites were local (*n* = 1), pulmonary (*n* = 3), or both (*n* = 1). Two out of 6 (33.3%) had local and pulmonary disease progression early on treatment before local control, and 4/6 had progression at end of treatment (Table 4 and Fig. 4).

**Fig. 5:**
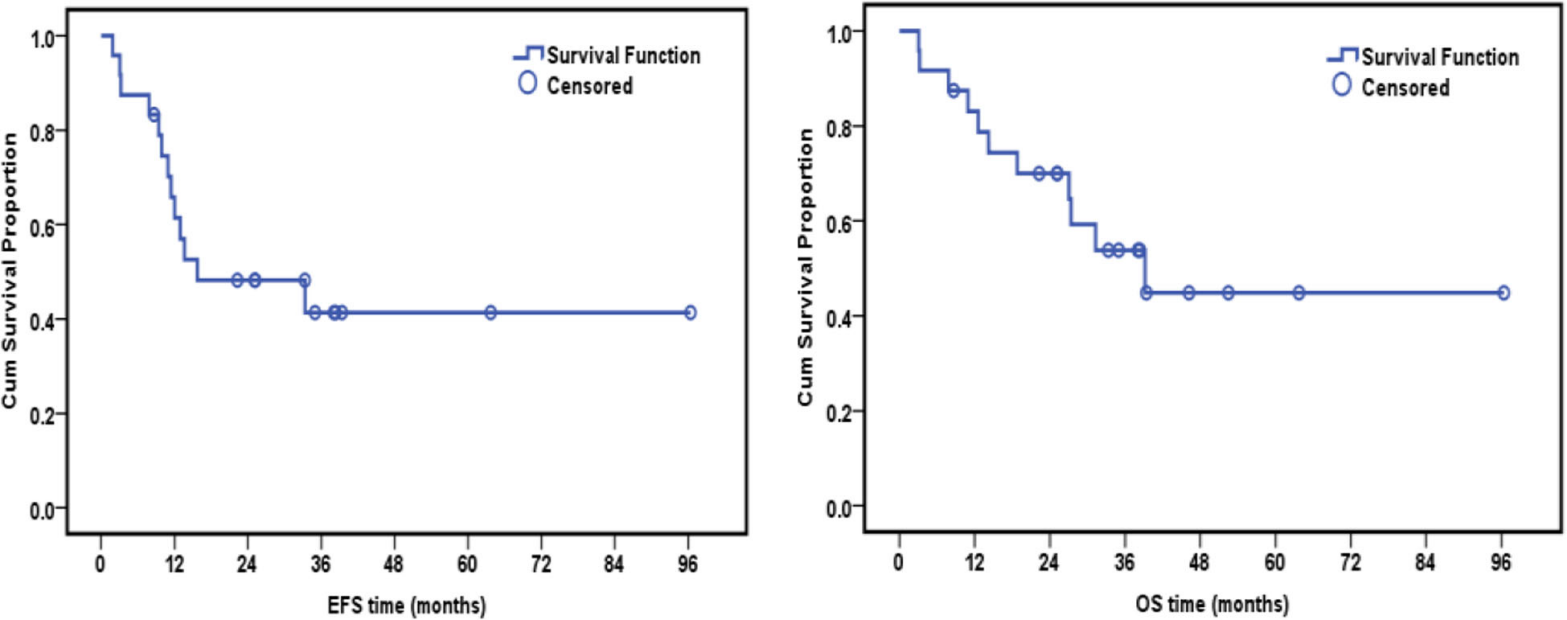
3-year event-free and overall survival of study patients

**Fig. 6:**
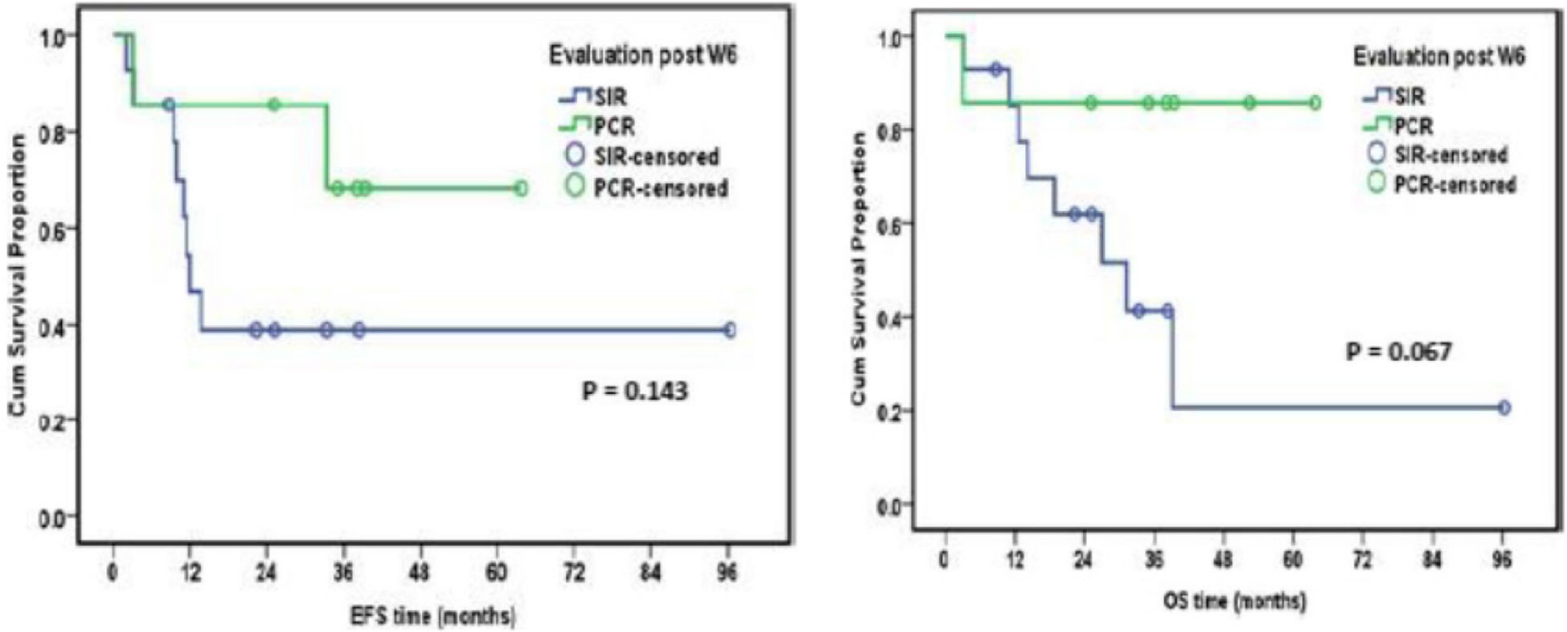
3-year event-free and overall survival by pulmonary response

**Fig. 7:**
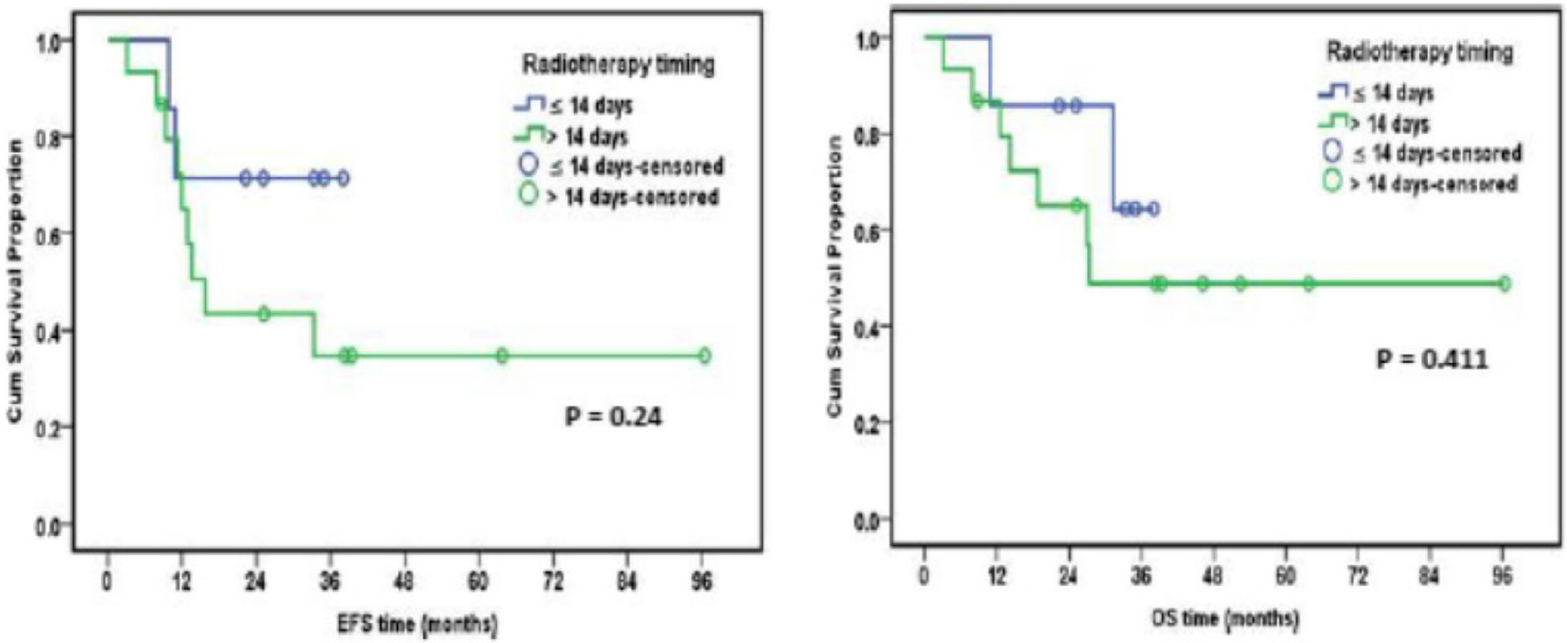
Event-free and overall survival by surgery radiotherapy interval

At a median follow-up of 27.2 months (range from 3.1–96.4 months), the 3-year EFS and 3-year OS were 48.2% and 54.2%, respectively (Fig. 5). Statistical analysis of survival outcome of study patients in correlation to their demographic and clinical features is summarized in Table 3. EFS and OS for patients who achieved RCR were higher than those who had SIR yet without statistical significance. Similarly, survival rates for patients who received their RTH within 14 days from surgery date were higher than those who received RTH later yet without statistical significance (Tables 3 and 4 and Figs. 6 and 7).

**Table 3:** Survival outcome of study patients in correlation to their demographic and clinical characteristics

Factors		*N*		3-year EFS	3-year OS	
			**%**	*P* value	%	*P* value
	All	24	48.2		53.8	
Age	< 5 years	15	60.0	0.065	57.1	0.633
	≥ 5 years	9	25.9		50.8	
Gender	Male	7	53.6	0.827	47.6	0.705
	Female	17	37.6		56.5	
Initial management	Biopsy/CTH	15	37.9	0.464	66.2	0.137
	Nephrectomy	9	44.4		44.4	
Initial metastases	Lung	21	47.5		57.6	
	Lung and liver	3	0	*	33.3	*
IVC involvement	Yes	3	33.3		66.7	
	No	21	45.5	*	51.3	*
Path. LNs involvement	Yes	6	50.0		33.3	
	No	9	19.5	0.657	34.6	0.655
Pulmonary response at W6	RCR	7	68.6		85.7	
	SIR	14	39.0	0.143	41.3	0.067
SRI	≤ 14 days	7	71.4		64.3	
	> 14 days	14	34.7	0.240	48.8	0.411

*P value could not be calculated due to small number of patients

*IVC* inferior vena cava, *LNs* lymph nodes, *SRI* surgery radiotherapy interval

**Table 4:** Details of management, response, and outcome of study patients

*Case NO.*	*Site of Mets*		*Local Stage*		*Initial Management*		*Response (W6)*		*Surgical Comp.*	*Pathology*			*RT*		*Adjuvant CTH*	*Events*			*0utcome*
	*Lung*	*Liver*		*Cause*	*Nephrectomy*	*CTH*	*Local*	*Met. Site*		*SM*	*LNs*	*Others*	*Local*	*Met. site*		*Lung*	*Local*	*Sepsis*	
*1*	*√*	*ˣ*	*3*	*Biopsy LNs*		*√*	*↓*	*SIR*	*ˣ*	*-VE*	*+VE*				*DD4A*	*®*			*Died*
*2*	*√*	*√*	*3*	*Biopsy*		*√*	*↓*	*↓*	*ˣ*	*-VE*	*Not assessed*				*Reg M*	*®*			*FU*
*3*	*√*	*ˣ*	*3*	*Biopsy IVC*		*√*	*↓*	*RCR*	*ˣ*	*-VE*	*-VE*				*DD4A*	*®*			*FU*
*4*	*√*	*ˣ*	*3*	*SM*	*√*		*Free*	*RCR*	*√*	*+VE*	*-VE*				*DD4A*			*√*	*Died*
*5*	*√*	*ˣ*	*3*	*Residual IVC*	*√*		*Free*	*SIR*	*ˣ*	*-VE*	*-VE*				*Reg M*	*P*			*Died*
*6*	*√*	*ˣ*	*3*	*Biopsy*		*√*	*↓*	*SIR*	*NO Nephrectomy*						*DD4A*	*P*	*P*		*Died*
*7*	*√*	*ˣ*	*1*		*√*		*Free*	*SIR*	*ˣ*	*-VE*	*-VE*				*Reg M*	*P*			*Died*
*8*	*√*	*√*	*3*	*Biopsy*		*√*	*↓*	*Free*	*ˣ*	*-VE*	*-VE*				*DD4A*		*®*		*Died*
*9*	*√*	*ˣ*	*3*	*Biopsy LNs*		*√*	*↓*	*SIR*	*√*	*-VE*	*+VE*				*Reg M*	*p*	*®*		*Died*
*10*	*√*	*ˣ*	*3*	*CTH*		*√*	*↓*	*SIR*	*ˣ*	*-VE*	*Not assessed*	*Blastem. pred.*			*DD4A*	*®*	*®*		*Died*
*11*	*√*	*ˣ*	*3*	*Biopsy*		*√*	*Stationary*	*SIR*	*Palliative Nephrectomy*			*Blastema. Pred.*			*ICE*	*P*	*P*		*Died*
*12*	*√*	*ˣ*	*3*	*T. Rupture LNs*	*√*		*Free*	*SIR*	*√*	*-VE*	*+VE*				*Reg M*	*P*	*®*		*Died*
*13*	*√*	*√*	*1*		*√*		*Free*	*↓*	*ˣ*	*-VE*	*-VE*				*Reg M*			*√*	*Died*
*14*	*√*	*ˣ*	*3*	*Biopsy*		*√*	*↓*	*SIR*	*ˣ*	*-VE*	*Not assessed*		*√*	*√*	*DD4A*				*FU*
*15*	*√*	*ˣ*	*3*	*Biopsy LNs*		*√*	*↓*	*SIR*	*ˣ*	*-VE*	*+VE*		*√*	*√*	*Reg M*				*FU*
*16*	*√*	*ˣ*	*3*	*Biopsy*		*√*	*↓*	*RCR*	*ˣ*	*-VE*	*Not assessed*		*√*	*ˣ*	*DD4A*				*FU*
*17*	*√*	*ˣ*	*3*	*Biopsy*		*√*	*↓*	*SIR*	*ˣ*	*-VE*	*-VE*		*√*	*√*	*Reg M*				*FU*
*18*	*√*	*ˣ*	*3*	*Biopsy*		*√*	*↓*	*SIR*	*ˣ*	*-VE*	*-VE*		*√*	*√*	*Reg M*				*FU*
*19*	*√*	*ˣ*	*3*	*Biopsy IVC*		*√*	*↓*	*SIR*	*ˣ*	*-VE*	*-VE*		*√*	*√*	*Reg M*				*FU*
*20*	*√*	*ˣ*	*3*	*Residual*	*√*		*Free*	*RCR*	*ˣ*	*-VE*	*Not assessed*		*√*	*ˣ*	*DD4A*				*FU*
*21*	*√*	*ˣ*	*3*	*Residual*	*√*		*Free*	*RCR*	*ˣ*	*+VE*	*Not assessed*		*√*	*√*	*DD4A*				*FU*
*22*	*√*	*ˣ*	*3*	*LNs*	*√*		*Free*	*SIR*	*√*	*-VE*	*+VE*		*√*	*√*	*Reg M*				*FU*
*23*	*√*	*ˣ*	*3*	*Residual*	*√*		*Free*	*RCR*	*ˣ*	*+VE*	*Not assessed*		*√*	*ˣ*	*DD4A*				*FU*
*24*	*√*	*ˣ*	*3*	*Biopsy LNs*		*√*	*Fee*	*RCR*	*ˣ*	*-VE*	*+VE*		*√*	*ˣ*	*DD4A*				*FU*

® relapse, P progression, ↓ regressive, LNs lymph nodes, IVC inferior vena cava, SM surgical margin, CTH chemotherapy, RT radiotherapy, FU follow-up, RCR rapid complete response, SIR slow incomplete response, Reg M 5-drug regimen, DD4A 3-drug regimen, ICE ifosfamide, carboplatin, and etoposide

## Discussion

Metastatic WT has a worse prognosis than localized disease and is less frequently seen in developed countries compared to developing world [6]. This study included 24 metastatic WT patients, constituting 23.3% of all WT patients presented to NCI, Egypt, during the study period. This percentage is similar to that reported in a similar period at Children Cancer Hospital of Egypt (CCHE) which reported 26% of their patients with WT as stage IV [7]. Reports from other developing African countries indicate a higher incidence of stage IV WT among their cohorts, with a reported incidence of 30.9%, 40%, and 42% in Nigeria, Kenya, and South Africa, respectively [8, 9]. While in developed countries, the incidence is relatively lower, with a reported incidence of 12%, 14.9%, and 17% in Children Oncology Group (COG), German study, and International Society of Pediatric Oncology (SIOP), respectively [10, 11]. This high incidence of advanced stages of WT noticed in developing countries could be attributed to several factors such as lack of awareness among caregivers and healthcare professionals regarding early presentations of WT, limited resources and poor access to health care that lead to late presentations, and advanced disease stage at diagnosis.

By the end of this study follow-up period, the 3-year EFS and OS were 48.2% and 54.2%, respectively. Comparable results were reported in similar studies—following SIOP guidelines—in South Africa with 5-year EFS and OS of 54.0% and 58.5%, respectively [12], and 5-year OS of 54.2% in another study following National Wilms Tumor Study Group (NWTSG) guidelines [13]. Another study conducted in Lebanon including only 9 patients treated for metastatic WT reported 3-year EFS of 55.6% and a 3-year OS of 67% [14]. Of note, the study carried out at CCHE reports a lower 3-year EFS of 18.2%, yet with higher 3-year OS of 66%. This might reflect a better salvage rates in their cohorts compared to ours [7].

On the other hand, international studies from developed countries report high survival rates for metastatic WT patients. In an Italian study; 5-year EFS and OS were 72.1% and 82.5%, respectively [15]. Another study in Japan reported a 5-year OS of 86.7% [16]. Also, higher rates were reported by COG and SIOP for stage IV WT patients. The SIOP 2001/GPOH trial reports a 5-year EFS and OS of 73% and 83%, respectively [17]. Similarly, NWTS-5 study reports a 5-year EFS and OS of 72% and 84%, respectively [18]. In 2018, a report from the COG AREN0533 study reported a 4-year EFS and OS of 85.4% and 95.6% respectively for stage IV WT patients (17).

There is some evidence that stage IV patients with local stage III disease have lower survival rates compared to local stage I and II patients. In SIOP 93-01, metastatic WT patients with local stage III had a significantly lower OS of 68% compared with local stage I and II patients (*P* < .001), and 5-year EFS rates were 82%, 83%, and 57% for patients with local stage I, II, and III disease, respectively (*P* < .001) [19]. Of note, all of our study patients except for 2 patients had local stage III, a fact that might—in part—explain the relatively lower survival rates of those patients.

Seven out of 9 patients (77.7%) who did an upfront nephrectomy had stage III local disease. This is relatively a high percentage of local stage III among up-front nephrectomy patients when compared to other studies. In UKW-3 trial, the immediate surgery group had 30% of tumors with stage III [20]. Notably, in the CTH and delayed surgery group, only 10% of tumors were stage III [20]. Moreover, in our study, surgical complications were reported in 3/9 patients (33.3%) who had up-front nephrectomy in comparison to one patient (7%) in the CTH and delayed nephrectomy group. A study of NWTSG—on 3335 children who underwent primary nephrectomy—reported surgical complications in 12.7% of patients [21]. The risk of tumor rupture was 15% in NWTSG for primary surgery and 3% for chemotherapy-pretreated cases in SIOP [20]. This was replicated by a randomized study in UK where surgical complications were observed in 14% of the up-front nephrectomy cohort while none of the neo-adjuvant CTH pretreated cases experienced any surgical complications [22]. A review of literature for the SIOP trials—since SIOP 1 trial until SIOP 2001—that focuses on the advantages of preoperative chemotherapy reported that surgery-related complications did not exceed 8% in the pretreated patients [23].

Up-front surgery versus delayed surgery had no statistical significance on outcome, despite high rate of surgical failure upfront. This is because of the poor outcome of the whole group and high rate of progression, and actually there is no evidence that local control can affect outcome of metastatic disease.

### Pulmonary and hepatic response

The COG, United Kingdom Children’s Cancer Study Group (UKCCSG), and SIOP trials evaluated different approaches to the management of children with stage IV pulmonary-only FHWT, seeking to avoid the use of whole-lung irradiation (WLI) because of its significant acute and long-term toxicity [24].

In this cohort, there were 2 cases with treatment-related mortality, specifically from severe sepsis. One case was in RCR after week 6 and was on lower doses of chemotherapy (3-drug regimen), while the other patient was on the higher doses of chemotherapy and went into severe neutropenia. Both cases had bacterial growth in their blood cultures together with pulmonary infection. This raises the importance of proper education and counselling for parents and caregivers regarding infection control measures and more importantly regarding early recognition of infectious complications in order to seek medical support when appropriate. Also, there should be close follow-up for the patient condition and their labs to give the proper supportive measures including empirical broad spectrum antibiotics and bone marrow stimulating factors if needed.

In our study, 7 patients (33%) of 21 patients with pulmonary-only FHWT achieved RCR. The SIOP 93-01 conducted a study on 234 patients with only pulmonary metastases, who received 6 weeks of neo-adjuvant 3-drug CTH, followed by nephrectomy. One hundred forty-eight (67.3%) of 220 patients who had complete data achieved RCR with combination CTH alone. An additional 37 patients required surgical resection of one or more pulmonary nodules to achieve RCR [18]. In contrast to the SIOP study, COG reported that only 133 of 292 patients (45.5%) with only pulmonary metastases were in RCR after 6 weeks of CTH [25], a finding possibly related to the administration of lower cumulative doses of doxorubicin during the pre-nephrectomy CTH period [18].

In our study, patients with RCR post 6 weeks of CTH had a better survival outcome compared to those with SIR, although without statistical significance. Of course, such results must be carefully interpreted with consideration to the very small number of studied patient and number of events. In the SIOP 93-01 study, 5-year EFS was 77% for those who achieved complete remission (CR) with CTH only, 84% for those who required surgical resection of residual nodules to achieve CR, 46% for patients with inoperable pulmonary metastases treated without WLI, and 50% for those with inoperable pulmonary metastases treated with WLI [18]. So, surgery should be encouraged for any accessible residual pulmonary nodules after neo-adjuvant CTH. In NWTSG-5, patients with stage IV FHWT with metastases limited to the lung had 5-year EFS of 85% in the setting of complete lung nodule response (CR) by day 70 versus 74% with incomplete lung nodule response, with all patients treated with 3-drug regimen [26].

A recent COG study reported that FHWT patients with week 6 lung nodule RCR treated without lung RT had 4-year EFS and OS of 79.5% and 96.1%, respectively. While for patients with SIR, 4-year EFS and OS estimates were 88.5% and 95.4%, respectively, with more intensified CTH (5-drug regimen) together with WLI [25].

As for patients with hepatic metastases, both the NWTS and the SIOP analyses show that patients with residual liver disease after treatment with CTH and/or RT that could be completely resected did well, suggesting that there is a role for complete surgical resection of residual metastases after adjuvant therapy [27].

### Local radiotherapy

As per protocol, irradiation should start at day 10–14 of therapy (with surgery done at day zero). In the current study, the median time to start radiotherapy after surgery was 19 days, with only 7/21 patients (33.3%) started their RT within 14 days from surgery date. This delay—in most cases—resulted from the burden of large numbers of patients relative to the capacity of RT machines. The National Cancer Database (NCDB) reports that for non-metastatic patients, surgery to radiotherapy interval ≤ 14 days correlates with improved overall survival. However, no association was noted for patients with metastases [28].

## Conclusions

The incidence of stage IV disease at presentation among our patients with WT is relatively higher than international groups, yet it is remarkably lower than other developing countries. This may reflect the variation in levels of awareness among caregivers and healthcare professionals as well as proportion of patients with delayed presentations rather than true ethnic variability. Abundance of local stage III disease among our patients, non-compliance to treatment protocols, and delay in starting irradiation might contribute to the relatively poorer outcome for our patients with metastatic WT compared to similar patients in other developed countries. Neo-adjuvant CTH and delayed nephrectomy approach is more suitable practice in the setup of a limited-resource center receiving large numbers of patients with advanced stage WT like our institute. Despite lack of statistical significance, the pulmonary response to neo-adjuvant CTH appears to be a strong predictive factor for outcome of patients with only pulmonary metastasis, and this needs further validation on a larger number of cases.

## Limitations of the study

This study was conducted retrospectively, and hence we were unable to justify the rational for some deviations from the therapy protocol. Some decisions related to field planning of RTH or upgrading treatment for slow responders were not complying with the protocol, and no clear explanation could be retrieved from patients’ records. Some fine details were difficult to verify in few cases as well, such as the reasons of delay in surgery or in starting RTH for some patients. The relatively small number of study patients precluded proper statistical inference for many studied variables.

## Data Availability

The datasets used and analyzed during the current study are available from the corresponding author on reasonable request.

## Abbreviations

CCHE: Children Cancer Hospital, Egypt
COG: Children’s Oncology Group
CR: Complete remission
CT: Computed tomography
CTH: Chemotherapy
EFS: Event-free survival
FHWT: Favorable histology Wilms’ tumor
GPOH: German Paediatric Oncology and Haematology Group
LOH: Loss of heterozygosity
NCDB: National Cancer Database
NCI: National Cancer Institute
NWTSG: National Wilms’ Tumor Study Group
OS: Overall survival
RCR: Rapid complete response
RT: Radiotherapy
SIOP: International Society of Paediatric Oncology
SIR: Slow incomplete response
UKCCSG: United Kingdom Children’s Cancer Study Group
WAI: Whole abdominal irradiation
WLI: Whole lung irradiation
WT: Wilms’ tumor
